# Interaction Effect Between Copy Number Variation in Salivary Amylase Locus (*AMY1*) and Starch Intake on Glucose Homeostasis in the Malmö Diet and Cancer Cohort

**DOI:** 10.3389/fnut.2020.598850

**Published:** 2021-01-07

**Authors:** Aida Koder Hamid, Johanna Andersson-Assarsson, Ulrika Ericson, Emily Sonestedt

**Affiliations:** ^1^Nutritional Epidemiology, Department of Clinical Sciences Malmö, Lund University, Malmö, Sweden; ^2^Wallenberg Laboratory, Department of Molecular and Clinical Medicine, Sahlgrenska Academy at University of Gothenburg, Gothenburg, Sweden; ^3^Diabetes and Cardiovascular Disease – Genetic Epidemiology, Department of Clinical Sciences Malmö, Lund University, Malmö, Sweden

**Keywords:** salivary amylase gene, copy number variation - CNV, dietary starch intake, plasma glucose, insulin resistance (HOMA-IR), epidemiology, type 2 diabetes

## Abstract

Salivary amylase initiates the digestion of starch and it has been hypothesized that salivary amylase may play a role in the development of insulin resistance and type 2 diabetes. The aim was to examine the interaction between copy number variation in the salivary amylase gene *AMY1* and starch intake. We studied 3,624 adults without diabetes or elevated blood glucose in the Malmö Diet Cancer cohort. We assessed the associations and interactions between starch intake, *AMY1* copies and glucose homeostasis traits (i.e., fasting plasma glucose, insulin and HOMA-IR) and risk of type 2 diabetes over an average of 18 follow-up years. *AMY1* copy number was not associated with glucose, insulin or HOMA-IR. We observed a significant interaction between starch intake and *AMY1* copies on insulin and HOMA-IR after adjusting for potential confounders (*p* < 0.05). The inverse association between starch intake and insulin and HOMA-IR was stronger in the group with 10 or more copies (*P*_trend_ < 0.001). In addition, we observed an inverse association between starch intake and type 2 diabetes in the group with 10 or more copies (*p*_trend_ = 0.003), but not in the other groups. This cross-sectional observational study suggests that *AMY1* copy numbers might interact with starch intake on glucose homeostasis traits. Interventional studies are required to determine whether individuals with high *AMY1* copy numbers may benefit from a high starch intake.

## Introduction

Salivary α-amylases cleaves starch molecules into smaller saccharides that are further digested by pancreatic α-amylases in the gut. Individuals with high salivary amylase levels have been shown to have faster oral starch digestion (1). Salivary amylase is encoded by the *AMY1* gene, which has gained interest because of the wide range of copy number variation (CNV) (2). In humans, the number of copies ranges from 2 to 17 (3, 4), which can be compared to pancreatic *AMY2A*, ranging from 0 to 4 copies, and *AMY2B*, ranging from 2 to 6 copies (3). Importantly, the *AMY1* copy numbers are proportional to the α-amylase content in the saliva (1, 5, 6). Consequently, individuals with low copy numbers of *AMY1* have lower salivary amylase levels and might therefore have a decreased capacity to metabolize starch into glucose. Despite the potential role of *AMY1* CNV, there are surprisingly few studies of its physiological role in humans. Studies have shown contradictory results regarding the postprandial glucose response after the consumption of starch (7–9). It has been hypothesized that salivary amylase may play a role in the development of insulin resistance and type 2 diabetes (7); however, observational studies in large cohorts investigating the association between *AMY1* CNV and glucose homeostasis traits are limited. One study found that low *AMY1* copy number indicates higher insulin resistance in asymptomatic adults (10), while other epidemiological studies could not replicate this finding (11, 12). Low *AMY1* copy numbers have been associated with an increased obesity risk in European and Asian populations (11–15). However, other studies could not replicate this inverse association with obesity in other European and East Asian populations (3, 4, 16). We have previously in a Swedish cohort found that *AMY1* CNV is associated with obesity only when considering the starch intake of individuals (17). In addition, high starch intake was associated with higher body mass index (BMI) among the group with the highest *AMY1* copy numbers. Individuals with high levels of salivary amylase may be better adapted to ingest starch, whereas individuals with low levels of salivary amylase may be at greater risk of insulin resistance and type 2 diabetes if having a habitually high starch intake (7). Thus, because of the suggested importance of salivary amylase levels for glucose homeostasis, we aimed to explore the association between *AMY1* CNV and levels of fasting plasma glucose, plasma insulin and estimated insulin resistance based on the homeostasis model assessment (HOMA-IR) and risk of type 2 diabetes and whether there is an interaction between *AMY1* CNV and starch intake on these traits.

## Materials and Methods

### Study Participants and Data Collection

The Malmö Diet Cancer (MDC) study is a population-based prospective cohort from southern Sweden with baseline examinations conducted between 1991 and 1996. All women born between 1923 and 1950 and men born between 1923 and 1945 living in Malmö were invited to participate (*n* = 74,138). The only exclusion criteria were limited Swedish language skills and mental incapacity. The participation rate was 41%, and 28,098 subjects completed all parts of the baseline examinations, with a higher participation rate in women than in men (17,035 females and 11,063 males). These baseline examinations included an extensive self-administered sociomedical questionnaire, anthropometric measurements and dietary assessment (18, 19). A random 50% of individuals recruited from 1991 to 94 were invited to participate in a cardiovascular subcohort (MDC-CV) with additional laboratory examinations, including fasting blood analyses. The ethical committee at Lund University approved the MDC study (LU 51-90), and all the participants provided written consent. Concentrations of glucose and insulin were measured from fasting whole blood samples. A conversion factor of 1.13 for glucose was used to convert whole blood values to standardized plasma values. Insulin resistance was estimated using HOMA-IR, which was calculated by the following equation: fasting plasma insulin (mU/L) × blood glucose (mmol/L)/22.5 (20). Of the subjects with *AMY1* CNV genotyped (*n* = 5,429), 5,099 had complete dietary data and 4,716 of those had glucose measured. After exclusion of individuals with diabetes at baseline (*n* = 225) and fasting glucose concentration 6.1 mmol/L or above (*n* = 867), our final study sample consisted of 3,624 individuals.

### Dietary Assessment

A three-part dietary history method was used based on a validated method specifically designed for the MDC study (21, 22). The self-administered portion was a combination of (I) a seven-day food diary and (II) a 168-item dietary questionnaire. The questionnaire assessed participants' meal patterns, estimated food intake frequencies and usual portion sizes using a photographic aid booklet. Thereafter, the participants were (III) interviewed for 1 h about food choices, food preparation practices, and portion sizes of the food recorded in the food diary. The information from the diary and the questionnaire was used to calculate average daily food intakes. Nutrient intakes were calculated using the MDC food and nutrition database developed for the MDC study, originating from the PC KOST2-93 of the Swedish National Food Agency. Additionally, seasonal variation in dietary reporting was accounted for using a separate variable. Starch intake was calculated by subtracting monosaccharaides and disaccharides from total carbohydrate intake. Starch was energy-adjusted using the nutrient density model, by calculating the percentage of starch from non-alcohol energy intake.

The relative validity of the dietary data has been examined and reported elsewhere (23). A slightly different diet method (130-item questionnaire and 2-week food records without the interview) was compared against a reference method of 18-day weighted food records for 1 year among 206 individuals living in Malmö from 1984 to 1985. The energy-adjusted correlation coefficients were calculated for carbohydrates (0.70 and 0.66 for men and women, respectively), fiber (0.69/0.74), fat (0.69/0.64) and protein (0.53/0.54).

### Genotyping and Copy Number Estimation

*AMY1* gene copy number was determined by droplet digital polymerase chain reaction (ddPCR) using the QX200 AutoDG ddPCR System (Bio-Rad Laboratories, Hercules, CA) following the manufacturer's instructions, as previously described in detail by Rukh et al. (17). A high-copy-number control (NA18972; HapMap DNA: 17 copies of *AMY1*) and two samples from female donors with known *AMY1* copy numbers (4 and 8 copies of *AMY1*) were included as controls in each run. We performed repeated runs of samples with high copy numbers and of a randomly selected subset of 10% of the samples. The plates were read on a Bio-Rad QX200 Droplet Reader, and copy numbers were calculated using QuantaSoft software version 1.7.4 (Bio-Rad Laboratories). Copy number of *AMY1* was divided into four groups: 1–4, 5–6, 7–9 and 10 and above copies.

### Type 2 Diabetes Ascertainment

Type 2 diabetes cases were identified through the end of the follow-up period (31 December 2014) using information from seven different registers [National Diabetes Register (24), the 2000 regional diabetes register (25), the local HbA1c register, the national inpatient register, the national outpatient register, the Swedish cause of death register (26), and the prescribed drug register] and information from MDC [baseline and rescreening (27)] and the Malmö Preventive Project (28). Individuals were considered to have diabetes if they were identified in any of these registers. This procedure has been described in detail elsewhere (26). For the analyses with type 2 diabetes, individuals with impaired fasting glucose were not excluded. In this study sample (*n* = 4,491), 471 incident T2D cases were identified during a mean follow-up of 18.3 years (82,568 years in total).

### Other Variables

A balance-beam scale was used to measure weight (kilograms) with subjects wearing light clothing and no shoes, and height (centimeters) was measured with a fixed stadiometer. BMI was defined as weight divided by height in square meters (kg/m^2^), and obesity was defined as BMI > 30 kg/m^2^. Educational level was divided into five categories: elementary, primary and secondary, upper secondary, further education without a degree and university degree. Smoking habits were categorized as current smokers (including irregular smoking), ex-smokers and never smokers. Alcohol consumption was divided into six categories. Zero consumers reported no consumption in the 7-day food diary or during the previous year in the questionnaire. The other individuals were divided into sex-specific quintiles based on the reported alcohol intake from the 7-day food diary, with the following cut-off values: 3.4, 9.1, 15.7, and 25.7 g/day (males) and 0.9, 4.3, 8.1, and 14.0 g/day (females). Leisure time physical activity levels were assessed according to 17 prespecified activities, with the option to add one more activity. Metabolic equivalent (MET) hours per week were calculated by multiplying the time spent on each activity in the four different seasons with an intensity factor, and the population were thereafter categorized into five groups (<7.5; 7.5–15; 15–25; 25–50; >50 MET hours per week).

### Statistical Analyses

IBM SPSS Statistics for Macintosh, version 24.0 (Armonk, NY: IBM Corp) was used for all the statistical analyses. Plasma insulin and HOMA-IR values were ln-transformed. Participant characteristics according to tertiles of starch intake and four categories of *AMY1* CNV was examined with ANOVA for continuous variables and chi-square test for categorical variables. A general linear model was used to examine associations between the exposure variables *AMY1* CNV (continuous) or starch intake (E%, continuous) and the outcome (dependent) variables of fasting glucose, insulin and HOMA-IR. In the basic model, we adjusted for age, sex and screening date. When examining the association between starch intake and outcome variables, we also adjusted for the following variables: leisure time physical activity, educational level, alcohol consumption, smoking habits, season of registry, energy intake, and BMI. These variables were selected for their potential association with either dietary intake or the outcome variables. We examined the interaction between starch intake and *AMY1* CNV on each outcome variable by including a multiplicative variable in the model with full adjustment. To interpret the findings from the interaction analyses, the association between *AMY1* CNV and outcome variables were examined in strata of starch intake tertiles, and the association between starch intake and outcome variables was examined in the strata of *AMY1* CNV groups. Means and 95% CIs for each outcome variable were calculated in strata of starch intake tertiles. We also ran the analyses separately for males and females.

Cox proportional hazard regression was used to calculate hazard ratios for type 2 diabetes incidence by *AMY1* CNV (adjusted for age, sex, and screening date) and starch intake (adjusted for age, sex, screening date, leisure time physical activity, educational level, alcohol consumption, smoking habits, season of registry, energy intake, and BMI). The interaction between starch intake and *AMY1* CNV on type 2 diabetes risk was assessed by introducing a multiplicative factor with continuous variables with the full adjusted model.

## Results

### Participant Characteristics

Our study sample of 3,624 individuals without diabetes and impaired fasting glucose included more females (*n* = 2,316; approximately 64%) than males (*n* = 1,308), with a mean age of 57 years (range 45–68 years). The participants' baseline characteristics according to tertiles of starch intake and groups of *AMY1* CNV are summarized in Tables 1, 2, respectively.

**Table 1:** Participant characteristics according to tertiles of starch intake among 3,624 individuals.

	**Starch intake tertiles**			
**Variables**	**Low (<22.6E%) (*n* = 1,208)**	**Medium (22-6-26.2E%) (*n* = 1,208)**	**High (>26.2E%) (*n* = 1,208)**	***P***
Starch intake (E%)	20.1 (2.0)	24.4 (1.1)	29.4 (3.2)	
Energy intake (kcal/day)	2,340 (656)	2,287 (643)	2,281 (672)	0.06
Fiber intake (g/1,000 kcal)	8.30 (2.54)	9.47 (2.49)	10.8 (3.0)	<0.001
Age (years)	57.6 (5.9)	56.9 (6.1)	57.2 (5.9)	0.01
BMI (kg/m^2^)	25.1 (3.7)	25.3 (3.8)	25.0 (3.3)	0.43
*AMY1* CNV	6.77 (2.43)	6.67 (2.48)	6.73 (2.43)	0.65
Fasting glucose (mmol/L)	5.36 (0.39)	5.38 (0.39)	5.38 (0.38)	0.43
Insulin (pmol/L)	41.7 (39.4)	40.3 (26.3)	40.0 (24.0)	0.32
HOMA-IR	1.48 (1.41)	1.44 (0.99)	1.42 (0.88)	0.37
Females (%)	891 (73.8%)	795 (65.8%)	630 (52.2%)	<0.001
University degree (%)	157 (13.0%)	156 (12.9%)	146 (12.1%)	0.10
Current smoker (%)	414 (34.3%)	271 (22.5%)	272 (22.5%)	<0.001
Low physical activity (%)	91 (7.6%)	94 (7.9%)	113 (9.4%)	0.74

*E%, energy percentage; BMI, body mass index, CNV, copy number variation*.

**Table 2:** Participant characteristics according to *AMY1* CNV groups among 3,624 individuals.

	***AMY1*** **CNV**				
	**1–4 copies (*n* = 737)**	**5–6 copies (*n* = 1,351)**	**7–9 copies (*n* = 932)**	**10 and above copies (*n* = 604)**	***P***
Females, *n* (%)	456 (63.1%)	878 (65.0%)	586 (62.9%)	387 (64.1%)	0.72
Age (y)	57.2 (6.0)	57.2 (5.9)	57.5 (6.0)	57.1 (6.0)	0.57
BMI (kg/m^2^)	25.0 (3.6)	25.1 (3.7)	25.1 (3.5)	25.3 (3.6)	0.44

*CNV, copy number variation*.

### Associations of *AMY1* CNV and Starch Intake With Glucose Homeostasis Traits

*AMY1* CNV was not associated with glucose, insulin or HOMA-IR; however, we observed an overall significant inverse association between starch intake and insulin and HOMA-IR, but not with glucose (Table 3). We observed a significant multiplicative interaction effect of starch intake and *AMY1* CNV on insulin (*P*-interaction = 0.03) and HOMA-IR (*P*-interaction = 0.04), but not on glucose (*P*-interaction = 0.99) (Table 4). Adjusting for fiber intake did not influence the results. *AMY1* CNV was positively associated with insulin and HOMA-IR only among individuals with low starch intake (<22.6 E%). In addition, the inverse association between starch intake and insulin and HOMA-IR was mainly observed among those with 10 or more copies of *AMY1*. In summary, the highest insulin levels and HOMA-IR were observed in the group that had highest number of *AMY1* copies and lowest starch intake (Table 4). When including all individuals without known diabetes (i.e., not restricting our study sample to those with normal fasting glucose concentrations, but also including those with prediabetes), we observed an interaction effect between starch intake and *AMY1* CNV on fasting glucose (*P* = 0.03). Similar as results for insulin and HOMA-IR, the negative association between starch intake and fasting glucose was only observed among those with 10 or more copies of *AMY1*.

**Table 3:** Associations between starch intake and fasting glucose, insulin and HOMA-IR^*^.

	**Starch intake tertiles**			
	**Low**	**Medium**	**High**	***p***
Fasting glucose	5.37 (5.35–5.40)	5.39 (5.36–5.41)	5.36 (5.33–5.38)	0.40
ln-insulin	3.61 (3.58–3.64)	3.59 (3.55–3.62)	3.57 (3.54–3.60)	0.003
ln-HOMA-IR	0.26 (0.23–0.30)	0.24 (0.21–0.27)	0.22 (0.19–0.25)	0.003

* *Means (95% CI) adjustment for age, sex, screening date, leisure time physical activity, educational level, alcohol consumption, smoking habits, season of registry, energy intake, and BMI*.

*HOMA-IR, homeostasis model assessment – insulin resistance*.

**Table 4:** Glucose, insulin and HOMA-IR (mean and 95% CI) by categories of starch intake and *AMY1* CNV.

	***AMY1*** **CNV**					
	**1–4 copies**	**5–6 copies**	**7–9 copies**	**10 and above copies**	P^*^	***P*-int^†^**
Glucose (mmol/L)						0.99
Low starch	5.35 (5.30–5.40)	5.37 (5.34–5.41)	5.35 (5.31–5.39)	5.38 (5.32–5.43)	0.75	
Medium starch	5.38 (5.33–5.43)	5.40 (5.36–5.43)	5.38 (5.33–5.42)	5.38 (5.33–5.44)	1.00	
High starch	5.39 (5.34–5.43)	5.38 (5.34–5.41)	5.37 (5.33–5.41)	5.38 (5.33–5.43)	0.72	
*P*^†^	0.71	0.60	0.61	0.77		
Ln-insulin [(ln)pmol/L]						0.03
Low starch	3.54 (3.47–3.60)	3.58 (3.53–3.62)	3.57 (3.51–3.63)	3.65 (3.58–3.72)	0.02	
Medium starch	3.53 (3.47–3.59)	3.57 (3.53–3.62)	3.55 (3.49–3.61)	3.57 (3.50–3.65)	0.92	
High starch	3.56 (3.50–3.62)	3.54 (3.49–3.59)	3.55 (3.49–3.61)	3.57 (3.50–3.64)	0.90	
*P*^†^	0.76	0.04	0.84	0.002		
Ln-HOMA-IR						0.04
Low starch	0.18 (0.11–0.25)	0.23 (0.18–0.28)	0.22 (0.16–0.28)	0.30 (0.23–0.37)	0.03	
Medium starch	0.18 (0.12–0.25)	0.23 (0.18–0.28)	0.20 (0.14–0.26)	0.23 (0.15–0.30)	0.91	
High starch	0.21 (0.15–0.28)	0.19 (0.14–0.24)	0.20 (0.14–0.26)	0.22 (0.15–0.29)	0.94	
*P*^†^	0.75	0.04	0.78	0.004		

* *Adjusted for age, sex and screening date*.

† *Adjusted for sex, age, screening date, leisure time physical activity, educational level, alcohol consumption, smoking habits, season of registry, energy intake, and BMI. CNV, copy number variation; HOMA-IR, homeostasis model assessment – insulin resistance*.

There was an interaction effect of starch intake and *AMY1* CNV on insulin and HOMA-IR for females (*p*-interaction = 0.005 and 0.008, respectively) but not for males (*P*-interaction = 0.78) (Table 5); however, the 3-way interactions between sex, *AMY1* CNV and starch intake was not statistically significant for neither insulin (*P* = 0.07) nor HOMA-IR (*P* = 0.09). In females, the inverse association between starch intake and insulin levels was only observed in the high *AMY1* CNV group.

**Table 5:** Association between starch intake and glucose, insulin and HOMA-IR (beta and SE) by categories of *AMY1* CNV categories.

	**Women**			**Men**		
	**ß (SE) per E% of starch^†^**	***P*^†^**	***P*-int^†^**	**ß (SE) per E% of starch^†^**	***P*^†^**	***P*-int^†^**
Glucose (mmol/L)			0.87			0.75
1–4 copies	0.002 (0.004)	0.63		−0.006 (0.005)	0.26	
5–6 copies	−0.006 (0.003)	0.06		0.006 (0.004)	0.12	
7–9 copies	−0.001 (0.004)	0.83		−0.002 (0.004)	0.67	
10 and above copies	−0.001 (0.005)	0.89		<0.001 (0.005)	0.94	
Ln-insulin [(ln)pmol/L]			0.005			0.78
1–4 copies	0.008 (0.005)	0.14		−0.011 (0.006)	0.07	
5–6 copies	−0.005 (0.004)	0.17		−0.007 (0.005)	0.16	
7–9 copies	0.001 (0.005)	0.84		−0.002 (0.006)	0.71	
10 and above copies	−0.013 (0.006)	0.03		−0.014 (0.006)	0.14	
Ln-HOMA-IR			0.008			0.78
1–4 copies	0.009 (0.006)	0.14		−0.012 (0.006)	0.06	
5–6 copies	−0.006 (0.004)	0.11		−0.006 (0.005)	0.22	
7–9 copies	0.001 (0.005)	0.87		−0.003 (0.006)	0.16	
10 and above copies	−0.013 (0.006)	0.04		−0.014 (0.007)	0.10	

† *Adjusted for age, screening date, leisure time physical activity, educational level, alcohol consumption, smoking habits, season of registry, energy intake, and BMI. CNV, copy number variation; HOMA-IR, homeostasis model assessment – insulin resistance*.

### Associations Between *AMY1* CNV or Starch Intake and Type 2 Diabetes Risk

We observed no statistically significant association between and *AMY1* CNV and type 2 diabetes risk (HR = 1.00, 95% CI: 0.97, 1.03 per additional copy; *P* = 0.82) or starch intake and type 2 diabetes risk (HR = 0.99, 95% CI 0.97, 1.00 per additional E% of starch intake; *P* = 0.09). Although there was no statistically significant interaction between *AMY1* CNV and starch intake (*P* = 0.30), we observed an inverse association between starch intake and type 2 diabetes among those with 10 or more copies of *AMY1* (*P* = 0.003), but not among those with fewer number of copies (Table 6).

**Table 6:** Association between starch intake and type 2 diabetes in strata of *AMY1* CNV categories^*^.

	***AMY1*** **CNV**			
	**1–4 copies**	**5–6 copies**	**7–9 copies**	**10 and above copies**
Hazard ratio (per additional E% of starch)	0.98 (0.94–1.02)	0.99 (0.96–1.01)	1.02 (0.99–1.06)	0.94 (0.89–0.98)
*P*	0.22	0.32	0.27	0.003

* *Adjustments for age, sex, screening date, leisure time physical activity, educational level, alcohol consumption, smoking habits, season of registry, energy intake, and BMI*.

*CNV, copy number variation, E%, energy percentage*.

## Discussion

In this study, we found that individuals with a higher dietary composition of starch had significantly lower insulin levels and insulin resistance (estimated by HOMA-IR). This inverse association tended to be stronger in the high *AMY1* copy number group. Furthermore, we found lower type 2 diabetes risk with higher starch intake in the group with high *AMY1* CNV.

Few studies have investigated the association between *AMY1* CNV and glucose homeostasis traits. Similar to our findings, there was a lack of association between *AMY1* CNV and HOMA-IR among French adults (11), Australian adults (8), and in a Finnish population aged 15–25 years (12). However, an inverse association between *AMY1* CNV and HOMA-IR was observed in asymptomatic Korean men with prediabetic conditions (10). The different results may be because of the different ethnic populations with different lifestyles, disease prevalence and genetic backgrounds. Another explanation of the conflicting results is the use of different CNV technologies. High-resolution CNV technologies, such as digital droplet PCR, have been shown to be more reliable for *AMY1* copy number estimation than real-time quantitative PCR (3, 4, 11); the latter was used in the Korean study.

According to our knowledge, this is the first observational study to investigate the fasting glucose and insulin levels according to starch intake while also taking *AMY1* CNV into account. However, there are a few studies investigating the postprandial glucose response to starchy foods among groups with either high or low *AMY1* copy numbers (7, 8). *AMY1* CNV is strongly correlated with the amount of salivary amylase activity (1, 5, 6), and higher salivary amylase activity promotes faster initial starch digestion (1, 7). Atkinson et al. hypothesized that individuals with high *AMY1* copy numbers would digest starchy foods faster and therefore have a higher glucose response than those with low copy numbers. Indeed, they found the group with the highest *AMY1* copies to have 15–40% higher postprandial glucose levels after consumption of 50 g available carbohydrates (8). This finding is in contrast to another study among 14 normal-weight individuals that found lower postprandial glucose levels among those with high amylase activity after ingestion of 50 g starch solution than individuals with low salivary amylase levels (7). Importantly, this finding was explained by earlier insulin release (i.e., cephalic phase insulin release) among the group with high amylase levels. These results suggested improved glucose homeostasis among individuals with high *AMY1* copy numbers.

In the present study, the highest fasting insulin, HOMA-IR and risk of type 2 diabetes was observed among individuals with a very high number of *AMY1* copies (10 copies or above) having the lowest starch intake. These finding could support the suggested improved glucose homeostasis of individuals with high *AMY1* copy numbers. Thus, if individuals with high *AMY1* copy numbers have a faster initial starch digestion with higher cephalic insulin release, high starch intake might be related to lower insulin resistance in this group. A high-starch diet is on average also higher in fiber, and fiber have been associated with lower risk of type 2 diabetes (29), which is supported by the negative association between starch intake (expressed in E%) and fasting insulin and HOMA-IR in the full study sample.

The inverse association between starch intake and insulin and HOMA-IR was strongest among individuals with 10 or more copies of *AMY1*. Similar association was observed between starch intake and glucose when not restricting the study sample to those with normal glucose concentrations. In this cross-sectional study, findings may be affected by reverse causation. We excluded individuals with prediabetes because they may have decreased their carbohydrate and starch consumption, either due to them being aware of their glucose levels or other health issues. However, many of those with prediabetes are asymptomatic and therefore not aware of their glucose levels. The narrow range, when only restricting the study sample to those below 6.1 mmol/L of glucose, may hamper the ability to discover the influence of *AMY1* CNV on fasting glucose. Although we found statistically significant differences for insulin and HOMA-IR, the differences are modest and maybe of limited clinical relevance. These findings should also be tested in an interventional setting.

In a study of overweight individuals, carriers of the A-allele of an *AMY1-AMY2* genotype, corresponding to high salivary amylase levels, had lower fasting glucose concentrations at baseline than the G-allele carriers (low salivary amylase levels) (30). However, individuals with the GG genotype (*n* = 380) showed lower fasting glucose after 6 months of the low-caloric intervention than those with the AA (*n* = 55) and GA (*n* = 257) genotype, independent of concurrent weight changes and baseline levels. We could not significantly connect *AMY1* CNV to a decreased type 2 diabetes risk, likely because of low power, but there is some evidence that supports a connection between salivary amylase activity and glucose homeostasis that is possibly affected by dietary changes.

Although *AMY1* CNV is strongly correlated with salivary amylase levels in saliva (1, 5, 6) and in plasma (11, 12), a large part of salivary amylase expression is due to other factors. *AMY1* CNV accounts for 11–35% of its protein expression (1, 5, 6), and the protein expression is only partly proportional to its enzymatic activity (5). Environmental factors such as diet, stress levels and circadian rhythm affect salivary amylase variation among individuals (2). For example, psychosocial stress has been shown to increase salivary amylase activity in saliva (31, 32), both in newly diagnosed type 2 diabetes patients and healthy controls (32). Additionally, patients with diabetes, both type 1 and type 2, seem to have lower serum amylase levels (33). Bonnefond et al. (11) found that plasma salivary amylase activity, but not *AMY1* CNV, was significantly associated with lower fasting plasma glucose levels and estimated β-cell function (HOMA-2B) in 3,504 French adults (11).

In several studies, the association between *AMY1* CNV and obesity traits has been sex-specific, i.e., found in only females with early-onset obesity (12) or in prepubertal boys (14). In the present study, the interaction between starch intake and *AMY1* CNV on the outcome was significant for only females. However, the inverse associations between starch intake and insulin and HOMA-IR were stronger in males and with similar effect sizes for all *AMY1* CNV groups, possibly because males have higher rates of insulin resistance than females (20).

The extended dietary data enabled us to estimate starch intake; however, a limitation of our study is the lack of intake of various types of starch. For example, we were unable to distinguish between resistant, rapidly and slowly digestible starch. Different starch types have different effects on glucose homeostasis; for example, rapidly digested starch leads to a rapid increase in plasma glucose followed by hypoglycemia (34). However, resistant starch results in an upregulation of important glycogenesis rate-limiting enzymes and insulin-induced genes, leading to an improved blood lipid composition, possibly due to an increased bacterial gut population in a rat model (35). Thus, future research on optimal carbohydrate measurement and subtype differentiation is important to understand how starch types affect the expression of *AMY1* experimentally and in observational studies.

In conclusion, we observed that individuals consuming a high-starch diet have lower fasting insulin levels and HOMA-IR. This association was stronger with higher *AMY1* copy numbers (specifically above 10 copies, 17% of this population). These results indicate that a high-starch diet may be beneficial for individuals with higher *AMY1* copy numbers.

## Data Availability Statement

The data analyzed in this study is subject to the following licenses/restrictions: The dataset for this article cannot be made publicly available because of ethical and legal restrictions. Requests to access these datasets should be directed to https://www.malmo-kohorter.lu.se/english.

## Ethics Statement

The studies involving human participants were reviewed and approved by the ethical committee at Lund University (LU 51-90). The participants provided their written informed consent to participate in this study.

## Author Contributions

ES formulated the research question and designed the study and had primary responsibility for the final content. AH and ES analyzed and interpreted the data. JA-A performed the genetic analyses. AH wrote the first draft of the manuscript. ES and UE provided critical review. All authors contributed to the article and approved the submitted version.

## Conflict of Interest

The authors declare that the research was conducted in the absence of any commercial or financial relationships that could be construed as a potential conflict of interest.
